# The genetic control of leaf allometry in the common bean, *Phaseolus vulgaris*

**DOI:** 10.1186/s12863-020-00838-2

**Published:** 2020-03-14

**Authors:** Miaomiao Zhang, Shilong Zhang, Meixia Ye, Libo Jiang, C. Eduardo Vallejos, Rongling Wu

**Affiliations:** 1 Center for Computational Biology, College of Biological Sciences and Technology, Beijing Forestry University, Beijing, 100083 China; 2 Department of Horticultural Sciences, University of Florida, Gainesville, FL 326511 USA; 3 Center for Statistical Genetics, The Pennsylvania State University, Hershey, PA 17033 USA

**Keywords:** Quantitative trait loci, Allometric equation, *Phaseolus vulgaris*, Leaf growth traits, Functional mapping

## Abstract

**Background:**

To maximize photosynthetic efficiency, plants have evolved a capacity by which leaf area scales allometrically with leaf mass through interactions with the environment. However, our understanding of genetic control of this allometric relationship remains limited.

**Results:**

We integrated allometric scaling laws expressed at static and ontogenetic levels into genetic mapping to identify the quantitative trait loci (QTLs) that mediate how leaf area scales with leaf mass and how such leaf allometry, under the control of these QTLs, varies as a response to environment change. A major QTL detected by the static model constantly affects the allometric growth of leaf area vs. leaf mass for the common bean (*Phaseolus vulgaris*) in two different environments. The ontogenetic model identified this QTL plus a few other QTLs that determine developmental trajectories of leaf allometry, whose expression is contingent heavily upon the environment.

**Conclusions:**

Our results gain new insight into the genetic mechanisms of how plants program their leaf morphogenesis to adapt to environmental perturbations.

## Background

Leaves play a central role in maintaining plants’ survival, growth and reproduction through the interception and transformation of solar energy into chemical energy to produce photosynthates [1–3]. Plants are equipped with a capacity to maximize photosynthetic efficiency by adjusting leaf characteristics such as surface area, mass, number, size, thickness, and nitrogen content, as well as their tradeoffs in changing environments [4]. Leaf surface area and leaf mass are two important features widely used as a proxy for leaf photosynthetic capacity [5]. Leaf area scales allometrically with leaf mass through complex interactions with the environments. The ratio of leaf surface area to leaf mass, i.e. specific leaf area (SLA), or its reciprocal (leaf mass per area, LMA) is an informative indicator of how plants adapt to different environments and how plants evolve new phenotypes [6–10].

The relationship between leaf mass and leaf area has been studied in several fields. As to the molecular mechanisms, Weraduwage et al. [11] detected CGR (*cotton Golgi-related*) -mediated pectin methylesterification that determines how carbon is allocated into leaf area and leaf mass through modulating the expansion and positioning of leaf cells in *Arabidopsis thaliana*. A model named Arabidopsis leaf area growth model was designed to simulate the plant growth process, the emphasis is on the effect of variation in C partitioning between leaf area growth and thickening [12]. Although there are studies that have mapped SLA in specific species [13, 14], our knowledge of genetic control of this allometric relationship of these two traits through genetic mapping remains scanty.

Several theories have been established through mathematical equations to describe the allometric relationship of two different traits, such as leaf area (cm^2^) and leaf dry weight (g) [15]. An allometric relationship is usually expressed by a power equation, expressed as

1a $$ A={\alpha M}^{\beta, } $$

or,

1b $$ \log A=\log \alpha +\beta\ \log M, $$

where *α* is the prefactor whose unit is cm^2^/g^*β*^ and *β* is the scaling exponent describing how leaf area (*A*) scales with leaf mass (*M*) [8, 16]. Leaf area vs. mass scaling predicts that SLA increases, stays constant, or decreases when *β* < 1.0, = 1.0, or > 1.0, respectively [15, 17–20].

The larger leaves have higher dry mass investment for per leaf area unit than the smaller ones to provide sufficient mechanical stability to adapt to unfavorable environments, as shown by the scaling exponent of leaf area vs. dry mass less than one (i.e. *β* < 1). This phenomenon can be explained by the “diminishing return” hypothesis (disproportionately smaller gains in total leaf surface area with increasing leaf mass or overall plant size would increase the “cost” of harvesting light) [15, 19]. However, the theoretical model of West, Brown, and Enquist proposes that the scaling exponent should be close to unity (i.e. *β* = 1.0) [17, 18] and that this scaling relationship should be insensitive to environmental changes [16]. In some cases, the scaling exponent was found to be greater than one, i.e. *β* > 1, which can be explained by the “network supply constraint” hypothesis (i.e. biological form and function can be predicted by the scaling properties of the vascular networks and the network is area-preserving across branching generations due to biomechanical constraints) [21]. Allometry as described in eq. (1) can be understood from static or developmental perspectives [22, 23]. If both leaf area and leaf mass are measured among different individuals at the same developmental stage or time point, it is called a static allometry. Yet, if both are measured on each individual over different developmental stage or series of time points, the relationship is called an ontogenetic allometry. Although these two types of allometry represent the outcome of a single biological process, namely, growth, the pathways by which they affect growth and the genetic mechanisms underlying their impact on growth may differ dramatically [23].

Wu et al. [24] pioneered a statistical model through genetic mapping that allows for the dissection of the genetic architecture of static allometry. The model can be used to identify QTLs that govern allometric relationships; this task can be accomplished through the incorporation of the power eq. (1) into a likelihood framework, which allows rigorous testing of QTL validity. The model was later modified for more biologically meaningful interpretations [25–28]. For a more complete approach, Li et al. [29] proposed a conceptual framework for mapping ontogenetic allometry by integrating allometry theory and functional mapping, a dynamic model for QTL mapping [30–32]. This framework allows testing the mode by which a QTL regulates allometric relationships over developmental time. Both static and ontogenetic allometry mapping models have been validated through the analysis of stem height vs. stem diameter growth in poplar and above- vs. below-ground biomass growth in soybean [25, 29]. Although the relationship between leaf area and leaf mass has been studied for decades [7, 14, 33, 34], how the allometric relationship varies with environmental change has never been validated by static and ontogenetic allometry mapping models simultaneously.

The common bean (*Phaseolus vulgaris* L.) is a legume that is widely grown in tropical and subtropical countries, where it represents an important source of proteins, carbohydrates, dietary fiber, vitamins, minerals, phytonutrients and antioxidants [35–37]. Numerous mapping studies have identified QTLs for time-to-flowering [38], node addition rate [36], and seed traits [39]. Although a few studies have begun to map leaf morphological traits in this species [39, 40], the genetic complexity of the mechanism that control allometric relationship of leaf area and dry weight has not been explored. The purpose of this study is to elucidate the genetic architecture of allometric scaling of leaf area with leaf mass in a recombinant inbred line (RIL) population of the common bean grown in two different environments, and examine how allometry QTLs change their expression over environment.

## Results

### Leaf area scales Allometrically with leaf mass in the RI family

**Fig. 1:**
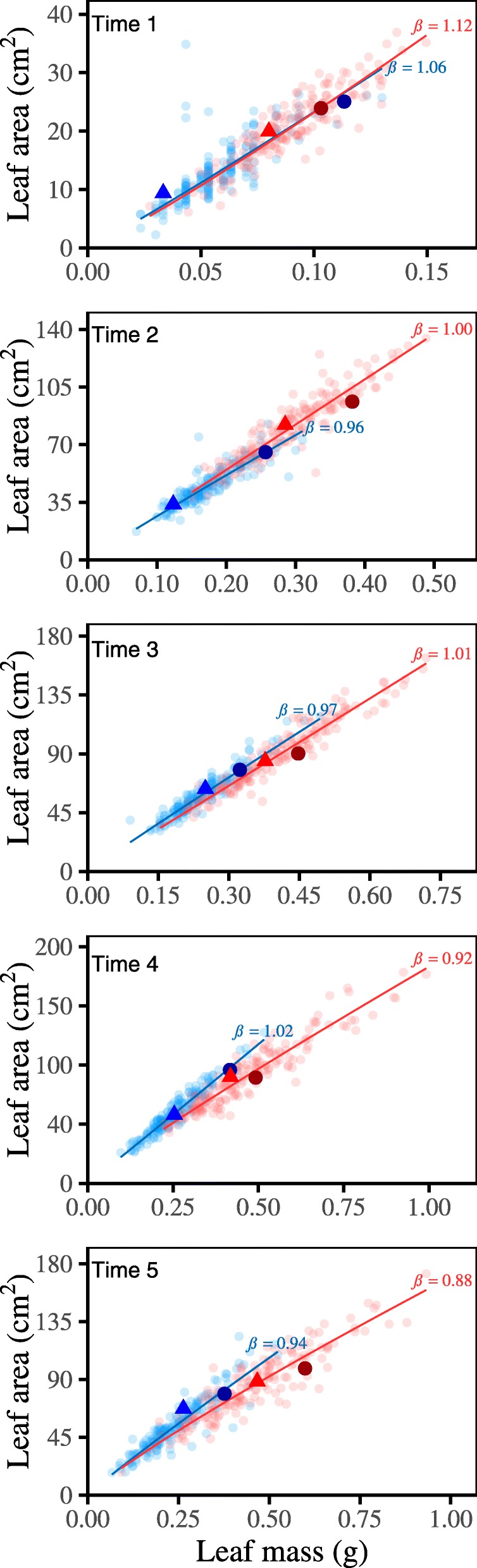
The static allometric scaling of leaf area vs. leaf mass at different time points 1–5. The static allometric scaling among the common bean RILs grown at Palmira (red) and Popayan (blue) are denoted by solid circles. The fitness of power equation to covarying leaf area and leaf mass data is indicated by curves at Palmira (red) and Popayan (blue), with the slopes of static allometry denoted by *β* estimates. The positions of two parents, Jamapa (triangles) and Calima (solid circles), in the static allometry of the RIL population, are indicated for two different locations

Plots of leaf area against leaf mass show that these characters follow the allometric scaling law in the parental and RIL genotypes of the common bean (Fig. 1). Accordingly, these characters can be modeled by the power eq. (1). Furthermore, this behavior was observed at the two environmentally different sites, Palmira (lower solar radiation, shorter day length, and higher temperature) and Popayan (higher solar radiation, longer day length, and lower temperature). The allometric scaling of the parental genotypes were different. The Calima parent consistently had larger and heavier leaves than the Jamapa parent, but these characteristics by the former decreased during development, especially in Popayan. An analysis of all genotypes showed that although allometric scaling was very similar at the two sites during the early developmental stage when leaves were not completely unfurled, the scaling begun to diverge soon afterward. Plants in Popayan grew a larger leaf area relative to leaf mass (*b* = 0.94) than those in Palmira (*b* = 0.88); however, leaves were larger and heavier in the latter than in the former. In summary, these plots indicated that allometric scaling of at least the first trifoliate changes with development and that this change depends on the environment. The plots in Fig. 1 also show extensive variation and transgressive segregation in the RIL population, suggesting the presence of genes that control many aspects of leaf growth and development and that each parent has alleles with contrasting contributions to the leaf traits.

### QTL control of static Allometry

The static model was used to map those QTLs that control leaf allometric scaling in the RIL family. The significant QTL regions and their peak QTLs detected by static model for each time point were shown in plot of Table 1 and Additional file 1. A consistent major QTL region was identified on chromosome 7 for all the time points except for time 1. The genetic effects of these QTLs on leaf mass and leaf area were different among QTLs and time points. The genetic effect of LSA7.2 in Palmira was greater than that in Popayan at time 3. All the peak QTL genetic effects were positive except LSA3.2 at time 4 located between 32.5–39.8 cM on chromosome 3.

**Table 1:** Peak quantitative trait loci (QTLs) located in significant QTL regions by static allometry model. QTLs were assigned to one of four categories: Leaf Ontogenetic Allometry (LOA), and Leaf Static Allometry (LSA)

Time Point	Peak QTL name	Chromosome (Position)	QTL region (cM)	Left-right marker	*Leaf mass (u*_*M*_ *= exp(u*_*z*_*))*			*Leaf area (u*_*A*_ *= exp(u*_*y*_*))*		
					CC	JJ	Genetic effect	CC	JJ	Genetic effect
Popayan										
Time 1	LSA3.1	3 (32.5)	32.5	DiM_3–18 - DiM_3–19	0.063 ± 0.002	0.052 ± 0.002	0.011	13.736 ± 0.596	12.061 ± 0.469	1.674
	LSA3.3	3 (74.2)	32.5–77.8	Bng216	0.063 ± 0.002	0.050 ± 0.002	0.013	13.736 ± 0.524	11.473 ± 0.581	2.263
	LSA9.1	9 (30.1)	29.3–31.1	DiM_9–22	0.063 ± 0.002	0.050 ± 0.002	0.013	14.585 ± 0.588	10.914 ± 0.460	3.672
Time 2	LSA7.5	7 (38.6)	29.5–56.0	DiM_7–8 - DiM_7–9	0.196 ± 0.005	0.151 ± 0.004	0.045	49.899 ± 1.394	39.646 ± 1.034	10.253
Time 3	LSA6.1	6 (49.2)	44.2–54.4	Bng183 - DiM_6–25	0.262 ± 0.008	0.219 ± 0.006	0.043	64.072 ± 1.982	51.935 ± 1.251	12.136
	LSA7.2	7 (35.9)	19.3–48.0	DiM_7–7 - DiM_7–8	0.267 ± 0.007	0.206 ± 0.005	0.061	64.716 ± 1.754	49.899 ± 1.304	14.817
Time 4	LSA4.1	4 (62.7)	60.6–63.8	DiM_4–18 - DiM_4–19	0.262 ± 0.009	0.223 ± 0.007	0.039	62.803 ± 2.100	50.400 ± 1.737	12.402
	LSA7.2	7 (35.9)	16.3–67.7	DiM_7–7-DiM_7–8	0.284 ± 0.007	0.208 ± 0.007	0.076	66.686 ± 1.672	47.465 ± 1.669	19.221
Time 5	LSA7.2	7 (35.9)	17.3–52.0	DiM_7–7-DiM_7–8	0.262 ± 0.010	0.190 ± 0.008	0.072	59.146 ± 2.027	42.948 ± 1.900	16.197
Palmira										
Time 1	LSA3.1	3 (32.5)	32.5	DiM_3–18 - DiM 3–19	0.096 ± 0.014	0.081 ± 0.015	0.015	22.421 ± 2.582	18.174 ± 2.698	4.247
Time 2	LSA7.3 (LOA7.2)	7 (36.6)	16.3–56.0	DiM_7–7 - DiM_7–8	0.32 ± 0.002	0.244 ± 0.002	0.074	89.121 ± 0.67	65.366 ± 0.614	23.440
Time 3	LSA7.2	7 (35.9)	11.4–56.0	DiM_7–7 - DiM_7–8	0.454 ± 0.007	0.326 ± 0.006	0.128	100.484 ± 1.737	70.81 ± 1.833	29.674
Time 4	LSA3.2	3 (33.8)	32.5–39.8	DiM_3–20	0.432 ± 0.016	0.449 ± 0.009	−0.017	80.64 ± 2.975	90.922 ± 1.933	−10.281
	LSA7.1	7 (33.5)	10.4–56.0	DiM_7–7 - DiM_7–8	0.522 ± 0.011	0.372 ± 0.009	0.150	100.484 ± 2.612	73.7 ± 2.09	26.784
Time 5	LSA7.2	7 (35.9)	14.3–60.9	DiM_7–7 - DiM_7–8	0.497 ± 0.018	0.368 ± 0.014	0.129	93.691 ± 3.118	69.408 ± 2.748	24.283

The QTL information at each time point by static allometry model for Palmira (PAL) and Popayan (POP) in Colombia was shown. Maximum likelihood estimates (MLEs) of parameters *u*_*M*_ and *u*_*A*_ (the power transformations of *u*_*z*_ and *u*_*y*_) and standard errors of the estimates for each QTL found to be different in the common bean (*Phaseolus vulgaris*) allometry relationship at two different sites, Palmira and Popayan. *u*_*M*_ and *u*_*A*_ are the genotypic values of leaf mass and leaf area for genotype CC and JJ. QTL region, the significant distribution range on chromosome. Peak QTL, the QTL located at the peak of the significant QTL region. Left-right marker, left marker and right marker on the both sides of peak QTL which located at the interval of two markers, particularly some peak QTLs located at a single marker

An overlapping QTL region on chromosome 7 was detected at both sites at times 2–4, and a major QTL located in this region LSA7.3 (also named LOA7.2 (LSA7.3)) between markers DIM_7–7 and DIM_7–8 was chosen for further analysis (Table 1; Additional files 1, 2). This QTL affected the allometric relationship of leaf area and leaf mass at both sites, but it appeared to be under developmental control. The mean values on leaf mass and area of LOA7.2 (LSA7.3) increased from time 1 to time 4, but decreased at time 5 for both sites, and the genetic effects showed similar patterns (Additional file 3). The mean values and genetic effect values for the same genotype were environment-dependent. The absolute values of environmental effects were getting bigger with time.

**Fig. 2:**
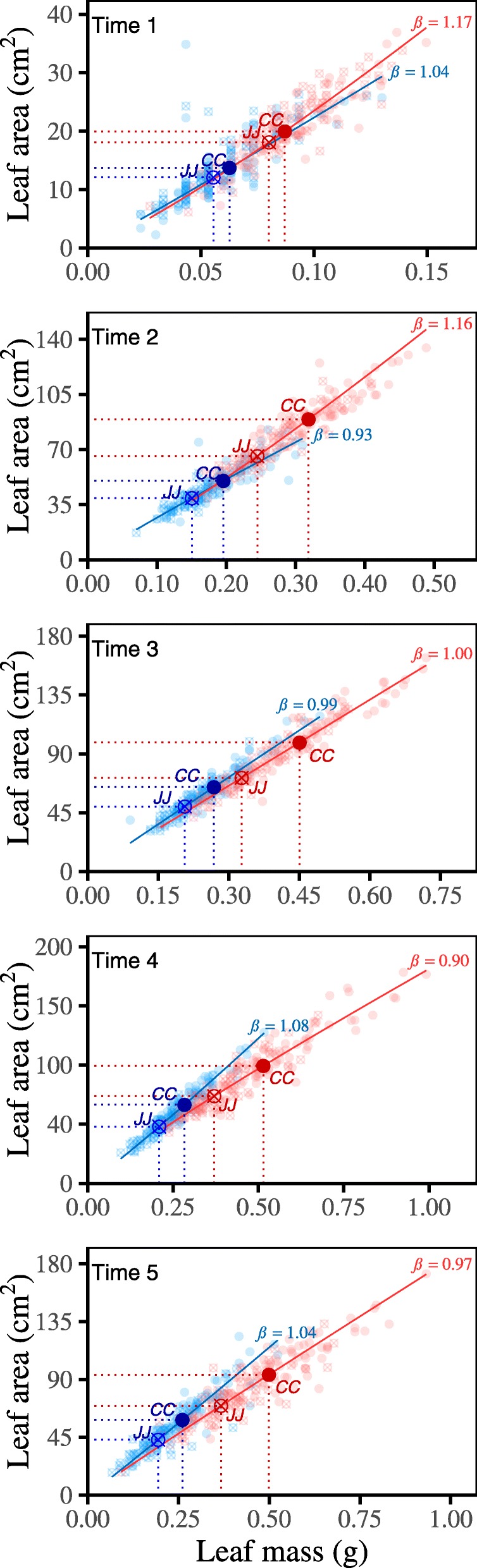
The static allometry of leaf area vs. leaf mass of LOA7.2 (LSA7.3). The static allometry for Jamapa-inherited genotype JJ (triangles) and Calima-inherited genotype CC (solid circles) of LOA7.2 (LSA7.3) at Palmira (red) and Popayan (blue) are shown at time points 1–5. The fitness of power equation to observed leaf area and mass data is indicated by curves at Palmira (red) and Popayan (blue), with the slopes of static allometry denoted by *β* estimates. The genotypic means of leaf area and leaf mass for JJ and CC at both locations are shown on the exponent curves

The effect of LOA7.2 (LSA7.3) is basically silent during the early stages of leaf development, and appears to be activated quickly after the leaf reaches maturity, but its effect decreases during the late stages of development (Fig. 2). Specifically, relative to the Jamapa LOA7.2 (LSA7.3) alleles (JJ), the Calima alleles (CC) are responsible for an increase in leaf area and leaf mass growth. Although LOA7.2 (LSA7.3) is a pleiotropic QTL for the two distinct environments, its effect varies between Palmira and Popayan, suggesting a remarkable QTL-environment interaction for leaf allometry (Fig. 2). The slope of leaf area-leaf mass allometry by LOA7.2 (LSA7.3) is generally consistent (*β* ≈ 1.04) over developmental time at Popayan, but it decreases (*β* = 1.17–0.97) as plants develop at Palmira. Under the control of LOA7.2 (LSA7.3), after the middle stage of plant development, leaves at Popayan tend to invest more energy to surface area growth relative to mass accumulation, with a greater extent than those at Palmira. Taken together with results from Fig. 1, LOA7.2 (LSA7.3) can be used to explain why leaf allometry varies in two different sites.

### How leaf area scales with leaf mass across developmental time

**Fig. 3:**
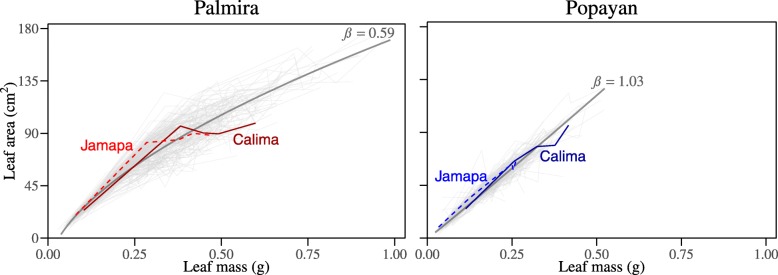
The ontogenetic allometry of leaf area vs. leaf mass for the common bean RILs grown at Palmira and Popayan. The ontogenetic allometry fitting of leaf area and leaf mass by eq. (5) *A*(*t*) *= αM*^*β*^(*t*) – *d* is indicated by curves (denoted by gray lines) at each location, with the slopes of ontogenetic allometry denoted by *β* estimates. The ontogenetic allometry of two parents Jamapa and Calima is also indicated

Despite tremendous variability among different RILs, the pattern by which leaf area growth covaries with leaf mass growth can be explained by the developmental allometry theory. Figure 3 illustrates the goodness-of-fit of the power eq. (8) to the mean curve of area-mass ontogenetic allometry among all RILs. The data clearly shows that the environment can have a significant effect on the ontogenetic control of allometric scaling of the leaf. First, RIL-RIL variation observed at Palmira was greater than at Popayan. Second, leaves at Palmira tend to accumulate more dry matter per unit of leaf area as they growth older than those at Popayan. Third, the slope of leaf area growth to leaf mass growth is close to unity (*β* = 1.01) (eq. 1a) at Popayan, whereas this *β* value is less than 0.90 at Palmira. This discrepancy may be due to different resource allocation patterns deployed under different environments in the leaves of these genotypes.

The two parents display different patterns of ontogenetic allometry. In Palmira, Jamapa’s slope of leaf area growth over leaf mass growth is greater than that of Calima from early to middle stages of growth (Fig. 3). This differential pattern shows that young Jamapa leaves expand their leaf area more than those of Calima for the same amount of accumulated mass. However, this pattern reverses from the middle to late stages of development. Such parent-dependent differences are much less pronounced in Popayan indicating the environmental sensitivity of this ontogenetic phenomenon. Pronounced transgressive segregation was found in ontogenetic allometry at both sites suggesting again a complex system.

### QTLs modulation of ontogenetic Allometry

We derived a dynamic allometry QTL mapping model based on the likelihood function (6), which integrates the ontogenetic allometric eq. (5) and functional mapping. We obtained the profiles of log-likelihood ratio test statistics at 2 cM intervals through the entire genome (Additional file 2) to scan for QTLs. Genome-wide significance tests allowed us to detect several significant QTL regions, including two regions on chromosome 7 (9.4–68.7 cM) and 6 (16.0–18.4 cM) at Popayan and two regions on chromosomes 7 (15.3–59.5 cM) and (23.9–25.6 cM) at Palmira (Table 2; Additional file 2). The genetic effects were different among different QTLs and between two sites for the common QTL LOA7.2 (LSA7.3). A major QTL named LOA7.2 that governs the scaling of leaf area over leaf mass throughout development at both sites. This QTL is located within markers DiM_7–7 and DiM_7–8 on chromosome 7, consistent with the location of the static allometry QTL LOA7.2 (LSA7.3) (Figs. S2, S3). This suggests that LOA7.2 (LSA7.3) is a major QTL triggering a remarkable impact on both static and ontogenetic allometry. In addition to LOA7.2 (LSA7.3), we detected two relatively small QTLs on chromosomes 6 (LOA6.1) and 9 (LOA9.1) that specifically affect ontogenetic allometry at Popayan and at Palmira, respectively.

**Table 2:** Peak quantitative trait loci (QTLs) located in significant QTL regions by ontogenetic allometry model. QTLs were assigned to one of four categories: Leaf Ontogenetic Allometry (LOA), and Leaf Static Allometry (LSA)

Peak QTL name	Chromosome (Position)	QTL region	left-right marker	*a*			*β*			*d*		
				CC	JJ	Genetic effect	CC	JJ	Genetic effect	CC	JJ	Genetic effect
Popayan												
LOA7.1	7 (33.9)	9.4–68.7	Bng042 - DiM_7–7	214.91 ± 5.45	231.05 ± 8.80	−16.14	0.86 ± 0.04	0.99 ± 0.04	−0.13	5.39 ± 2.13	−2.43 ± 1.65	7.82
LOA7.2 (LSA7.3)	7 (36.6)	9.4–68.7	DiM_7–7 - DiM_7–8	216.01 ± 5.24	202.32 ± 7.9	13.69	0.87 ± 0.04	0.85 ± 0.05	0.02	4.24 ± 2.05	3.59 ± 2.29	0.65
LOA6.1	6 (17.4)	16.0–18.4	DiM_6–15	218.58 ± 5.52	211.49 ± 6.37	7.09	0.90 ± 0.04	0.88 ± 0.04	0.02	3.57 ± 2.14	3.21 ± 1.88	0.36
Palmira												
LOA7.1	7 (33.9)	15.3–59.5	Bng042 - DiM_7–7	209.82 ± 9.96	178.33 ± 3.91	31.49	0.45 ± 0.05	0.63 ± 0.06	−0.18	48.58 ± 12.2	18.05 ± 6.68	30.53
LOA7.2 (LSA7.3)	7 (36.6)	15.3–59.5	DiM_7–7 - DiM_7–8	207.04 ± 5.16	178.41 ± 4.27	28.63	0.47 ± 0.03	0.62 ± 0.05	−0.15	44.57 ± 11.12	18.05 ± 5.83	26.52
LOA9.1	9 (25.6)	23.9–25.6	DiM_9–17	200.10 ± 8.12	185.19 ± 3.85	14.91	0.50 ± 0.05	0.64 ± 0.04	−0.14	36.60 ± 10.49	18.41 ± 5.61	18.19

The QTL information by ontogenetic allometry model for Palmira (PAL) and Popayan (POP) in Colombia was shown. Maximum likelihood estimates (MLEs) of parameters (*a*, *β*, and *d*) and standard errors of the estimates for each QTL found to be different in the common bean (*Phaseolus vulgaris*) allometry relationship at two different sites, Palmira and Popayan. On chromosome 7, LOA7.1 and LOA7.2 (LSA7.3) were the peak QTLs for POP and PAL respectively, the information of these two QTLs for both sites were listed. QTL region, the significant distribution range on chromosome. Peak QTL, the QTL located at the peak of the significant QTL region. Left-right marker, left marker and right marker on the both sides of peak QTL which located at the interval of two markers, particularly some peak QTLs located at single marker

**Fig. 4:**
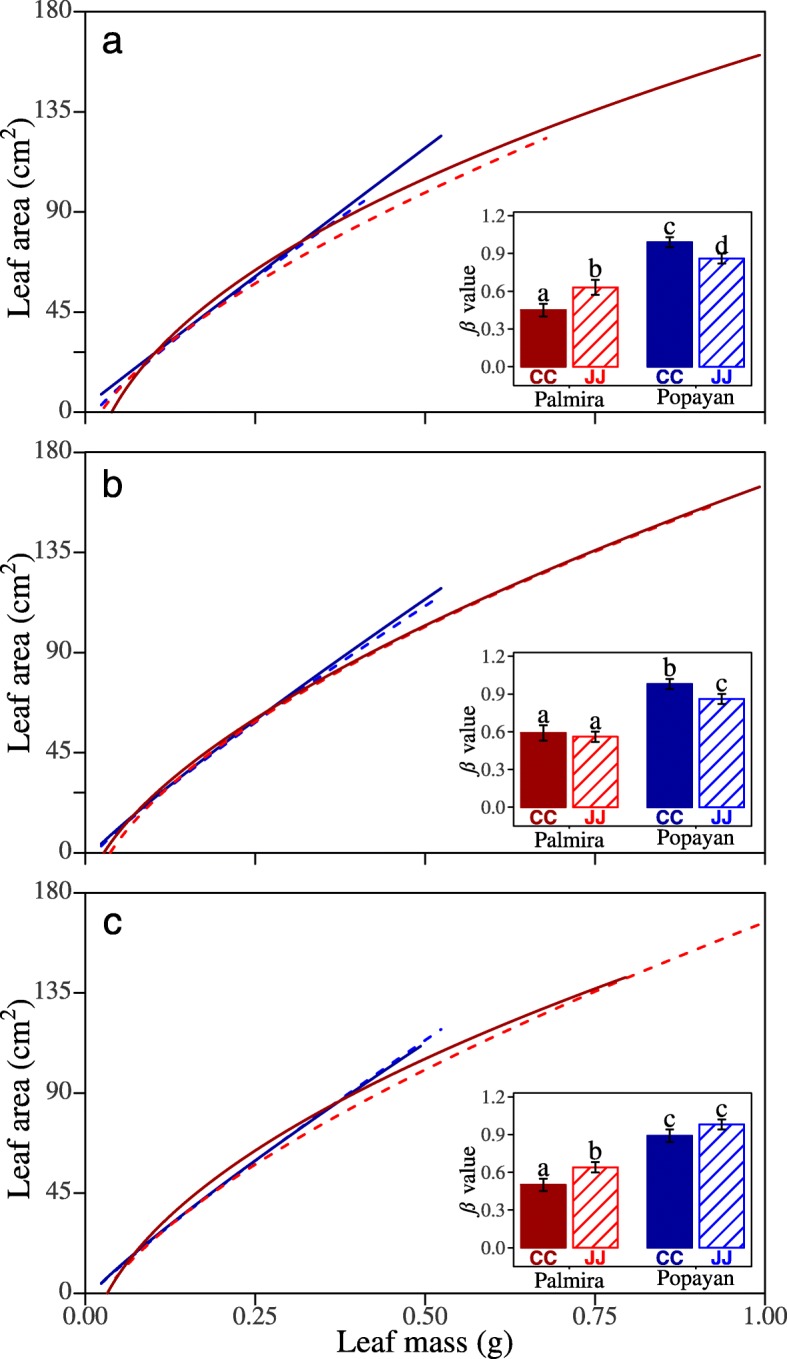
The ontogenetic allometry of leaf area vs. leaf mass for different QTL. Jamapa-inherited genotype JJ (slash curve) and Calima-inherited genotype CC (solid curve) at LOA7.2 (LSA7.3) (**a**), at Popayan-specific QTL LOA6.1 on chromosome 6 (**b**), and at Palmira-specific QTL LOA9.1 on chromosome 9 (**c**) for Palmira (red) and Popayan (blue). The estimated slopes of ontogenetic allometry (*β*) for different genotypes at different locations are indicated at the right lower corner of the plot. Significance tests of *β* differences are shown by different letters

We further characterized the genotype-dependent mode of action of locus (LOA7.2 (LSA7.3), LOA6.1 and LOA9.1) and their environmental dependencies as observed by the changes observed from Palmira to Popayan. The allometric parameter values and genetic effects of the same QTL were different between two sites. The absolute values of environmental effects of each parameter at Palmira were greater than that at Popayan (Additional file 3). Genotype CC for LOA7.2 (LSA7.3) displays a greater slope of allometric change over time, leading to larger leaf area and leaf mass, than genotype JJ at Palmira (Fig. 4a). This QTL has an inverse pattern of genetic effect at Popayan, i.e. genotype JJ has a greater slope than CC. The phenotypic difference of the same genotype expressed in different environments is called phenotypic plasticity. Genotype JJ has greater phenotypic plasticity in the allometric slope than genotype CC. At LOA6.1 which only significant only at Popayan, genotype CC has a greater slope than JJ at Popayan, and the allometry growth curves almost coincided (Fig. 4b). The differences between genetic effects of two sites on LOA6.1 were smaller than that on the other two QTLs. LOA9.1 on chromosome 9 only affects ontogenetic allometry at Palmira, at which genotype JJ has a greater slope and larger leaf area and leaf mass than CC (Fig. 4c). These two QTLs are environment-specific, triggering their effects on ontogenetic allometry depending on where the plants are grown. After function annotation, there were candidate genes coded hypothetical proteins that located at the most of the significant QTLs detected by both models (Additional file 3).

## Discussion

Leaves, the aboveground resource-acquiring organ of plants, play a pivotal role in maintaining plant growth and development [2, 3]. The allometric relationship between leaf area and leaf mass reflects the pattern of carbon allocation and plants’ resource-acquiring strategy [9, 10]. Ecological research has endeavored long to understand the role leaf allometry plays in evolution [5, 10, 11, 41], and the genotypic variation of specific allometric relationship between leaf area and mass has been extensively measured [7, 14, 33, 34]. Despite of these efforts, our knowledge of the genetic control of the allometric relationship of leaf area and leaf mass remains limited. More importantly, how the allometric relationships of these two leaf traits vary with environmental change has never been validated by static and ontogenetic allometry mapping models simultaneously.

The covariation of different traits is thought to obey allometric scaling laws that can be interpreted from fundamental principles of biophysics and biochemistry [28]. Allometry is very well studied and the genetics behind various allometric relationships have been examined across a variety of taxa [25, 29, 41]. In this article, we used two alternative models to analyze the genetic architecture of allometric scaling between leaf area and leaf mass in the common bean. The static model characterizes how one trait scales with other traits at the same developmental stage across all individuals, whereas the ontogenetic model specifies the allometric relationship of one trait with other traits across differential times [23]. Both static allometry and ontogenetic allometry have been used to address the fundamental question of whether microevolutionary processes can explain patterns of macroevolution, a still unsolved question in evolutionary biology [42]. Mathematical analysis showed that these two processes differ from each other with the extent of difference affected by the covariance between trait value and the ontogenetic allometric slope [23].

As a valuable legume due to its higher nitrogen fixing capacity [37], the ecophysiological studies of the common bean, aimed to explore its allometry of leaf area and leaf mass, are particularly interesting to plant breeders. We found that the static allometry of these two traits varies in its slope among different developmental times (Fig. 1). The formula reveals that investment of dry mass to the light-capturing surface per unit is following the scaling exponent of leaf area vs. leaf mass. Compared with those at young stages, leaves at old stages tend to invest more energy to structural and defense tissues, with increasing dry mass per leaf area [5, 43, 44]. At POP with low temperature and high light, the scaling exponent of leaf area and mass was closed to unity, suggesting that the dry mass kept the same speed as leaf area (*β* = 1.03, Fig. 3). As to the high temperature and weak light environment at PAL, the common bean allocated more carbon to expand leaf area for light harvesting at early growth stage (*β* = 1.12, Fig. 1), which can be explained by the “network supply constraint” hypothesis [21]. However, at late growth stage when the leaf area reached its maximum value, the leaves invested higher dry mass for per leaf area unit, as shown by the scaling exponent of less than one (*β* = 0.59, Fig. 3). This phenomenon can be explained by the “diminishing return” hypothesis [15, 19].

The allometry of these two traits varied in parameter values, genetic and environmental effects on the same QTL between different environments (Additional file 3). Using the same RIL mapping population, two studies have also detected the heterochrony QTLs that were pleiotropically expressed at PAL and POP [45, 46]. The former explored the role of these QTLs in influencing the dynamic growth curves of leaf area and leaf mass using four heterochronic parameters [45], while the latter implemented a bivariate statistical procedure to identify QTLs by estimating growth parameters of the two leaf traits and incorporating these parameters into a mapping framework [46]. Different from these studies, we focused more on the genetic mechanism underlying allometric relationships between the two leaf traits by introducing allometric model in genetic mapping framework, perspectivity from the union of ecology and genetics. The ontogenetic allometry at warmer PAL can be better explained by the “diminishing return” hypothesis; that is, as leaves grow, the area increases more slowly than the mass (Fig. 3). At cooler POP, the slope of the ontogenetic allometry is close to one, suggesting that a mix of the “diminishing return” and “network supply constraint” hypotheses may influence the scaling of leaf form. The results obtained from the analysis of environment-induced differences confirms the argument that static and ontogenetic allometry undertakes different physiological mechanisms [23]. Comparing the allometry relationship of PAL and POP (Fig. 1; Fig. 3), plants at PAL with weak light (lower solar radiation and shorter day length) and higher temperature adopted different carbon allocation strategy on leaf growth. The common bean tended to allocate nutrient substance to expanded leaf area for improving light capture efficiency under low light condition at early growth stage. After the middle growth stage, leaf area could meet the light capture demand, the plant began to increase blade thickness or dry weight. While common bean growing at POP with intense sunlight but lower temperature distributed more carbon to blade thickness (leaf mass) against the cold at the beginning of growth stage than the plant at PAL, with the two leaf traits increasing in the same pace.

We implemented static and ontogenetic allometry into a genetic mapping setting, which enables the characterization of the genetic origin of allometry. Specific QTLs mapped by this strategy could facilitate our understanding of how covariation between different traits drives the phenotypic evolution of species. We have found specific sets of QTLs for the static allometry and ontogenetic allometry of leaf area vs. leaf mass in the common bean. The genetic effects and environmental effects of the QTLs were different between two sites, implying the pleiotropic effects of the QTLs (Additional file 3). There was a major QTL region on chromosome 7 associated with allometry relationship of two leaf traits by both genetic mapping models. One QTL located on this region was found to exert a major effect on how these two traits scale among different individuals and also how they scale over developmental time. The QTL LOA7.2 (LSA7.3) was the most significant one that has been detected to affect leaf area-leaf mass allometry. The QTL region on chromosome 7 we detected overlapped with those found in *P. vulgaris* that associated with shoot, root, seed and disease resistance traits [36, 47–51]*.* This QTL region also included Sho7.1, Sho7.2, Sho 7.3, and swg7.1 that closely related to leaf area, length, width, and dry weight of *P. vulgaris* [51], because LOA7.2 (LSA7.3) was related to leaf growth. Besides, this QTL region was also found to be associated with pod width, pod thickness [48], yield, pod harvest index, seed weight, pod weight [50], and seed nutrient accumulation [47], indicating that LOA7.2 (LSA7.3) may also play an important role in yield production and implying a strong correlation between leaf photosynthesis and yield production. It is interesting to note that LOA7.2 (LSA7.3) resides within the same location of a major pleiotropic QTL (named *pleioQTL*) that affects growth parameters of leaf area and leaf mass for the common bean grown in two different environments by two models. The major QTL region detected by static and ontogeny models at both sites in our study was almost overlapping with the region found in the former study. The peak QTL LOA7.2 (LSA7.3) of this region located between DiM_7–7–DiM_7–8, the same as *LeafG1* and QTL LOA6.1 located at DiM_6–15 on chromosome was also very close to *LeafG1* (Position: DiM_6–15–Bng088) detected by Jiang et al. The QTLs LOA7.2 (LSA7.3) /*LeafG1* and LOA6.1*/LeafG*2 exerted pleiotropic effects on different traits and displayed environmental pleiotropy on the same trait between different environments [45]. Likewise, they also acted on the allometric growth relationship of these two leaf traits and displayed environmental pleiotropy as well in current study. A further genetic manipulation, such as cloning, may shed light on the molecular basis of how this QTL universally governs multiple developmental aspects of leaf growth.

## Conclusions

This study provides a contrasting picture of genetic architecture for static and ontogenetic allometry relationships between two different environments. We found a common QTL region and analysed the effect of a major pleiotropic QTL located in this region. Also, we found a couple of minor QTLs for leaf allometry. These QTLs are highly specific to the type of allometry, static vs. ontogenetic, to developmental time, early vs. late, and to environment, warmer and low-light vs. cooler and high-light. Alleles at these QTLs inheriting from parent Calima or Jamapa may contribute favorably or unfavorably to the increasing allometric slope of leaf area vs. leaf mass, one of the driving forces that cause transgressive segregates in the cross population. These recombinants, detected by our allometry mapping strategy, provide a fuel for plants to adapt to their new environments by adjusting the relative growth of leaf area and leaf mass. Overall, our findings facilitate a better understanding of the genetic mechanisms underlying plants programing their leaf morphogenesis in adaptation to environmental changes.

## Methods

### Mapping population and genotyping

A recombinant inbred line (RIL) population was generated from the cross between the Mesoamerican bean cultivar Jamapa and the Andean cultivar Calima of *Phaseolus vulgaris* L. The RI family comprises 173 lines, which was propagated by single seed descent and in bulk afterwards to the F_11:14_ generation. The nuclear DNA was extracted from leaf of each RI line, and then *PstI* GBS libraries were prepared and submitted for genotyping by sequencing in the Illumina HiSeq platform. Sequence data were processed by multiple bioinformatics tools to obtain SNPs. Linkage map was constructed with 513 unique loci covering 943 cM, which included 442 SNP loci (DiM), 66 RFLP-based markers, three soybean-derived SNP markers, and two phenotypic marker loci for this RILs population. The two parents and RILs were genotyped for 513 molecular markers located on 11 linkage groups each covering a common bean chromosome [40].

### Experimental design and data collection

The mapping population (including both parents) was planted at two sites, Palmira and Popayan in southwestern Colombia during 2011–2012. These two sites have different temperature regimes [45]. In Palmira, the mean air temperature ranges from 19.5 to 28.8 °C; solar radiation, 14.7 MJ m^− 2^ d^− 1^; day length, 15.6 h; and growing season, from 11 Nov 2011 to Jan 2012. In Popayan, the mean air temperature ranges from 13.7 to 25.5 °C; solar radiation, 11.8 MJ m^− 2^ d^− 1^; day length, 12.1 h; and growing season, from 23 Mar 2012 to Jun 2012. A randomized complete block row-column design with three replicates (six for each parent) was employed at each site. Each RIL plot had between 30 and 50 plants [46]. One plant was harvested weekly from each RIL plot and from all three replicates. The first five leaves were harvested and measured independently for leaf area and mass (dry weight). The weekly leaf samplings started soon after the plants reached stage V0 (i.e. the most of leaves are fully-expanded) and ended when the plants reached stage R1 (i.e. the time at first anthesis). Data of the first trifoliate leaf at each time point were employed to obtain the mean of three replicates for each RIL for QTL mapping. The combined area of the leaflets of each leaf was measured using a Li-Cor® LI-3100C area meter after harvest. Leaf blades were dried at 65 °C in a drying oven for 3 days. Leaves were equilibrated to room temperature after removing them from the oven before weighing them on a balance with a 10^− 3^ g resolution. We obtained data on leaf area and leaf mass of 173 lines at five time points from each site.

### Statistical modeling

#### Mapping static allometry

Suppose there is a mapping population of *n* recombinant inbred lines (RILs) in which there are two alternative homozygous genotypes at each marker. We are interested in the allometric covariation of leaf area and leaf mass, which can be described by a power eq. (1a) for each time point (time 1, time 2, time 3, time 4, and time 5). Let *y*_*i*_ and *z*_*i*_ denote log-transformed values of leaf area and leaf mass measured at a time point for RIL *i* (*i* = 1, …, *n*), respectively. Consider a QTL with two genotypes *QQ* (coded as 1) and *qq* (coded as 2). Let *μ*_1*y*_ and *μ*_1*z*_ denote the genotypic values of leaf area and leaf mass for genotype *AA*, and *μ*_2*y*_ and *μ*_2*z*_ denote the genotypic value of leaf area and leaf mass for genotype *aa*, respectively. If this QTL affects leaf allometry, we can establish the following relationships from equation (1b):

2a $$ {\mu}_{1y}=b+\beta {\mu}_{1z} $$

2b $$ {\mu}_{2y}=b+\beta {\mu}_{2z} $$

by letting *b* = log*α*. The genetic effects of this QTL on leaf mass and leaf area are calculated as *a*_*z*_ = *μ*_1*z*_ − *μ*_2*z*_ and *a*_*y*_ = *μ*_1*y*_ − *μ*_2*y*_ = *βa*_*z*_, respectively. The intercept of leaf area regressed on leaf mass is estimated as $b=\frac{1}{2}\left[\left({\mu}_{1y}+{\mu}_{2y}\right)-\beta \left({\mu}_{1z}+{\mu}_{2z}\right)\right]$.

Using this QTL’s information, we formulate a bivariate likelihood for two leaf traits based on a mixture model, expressed as

3 $$ L\left(\varPhi |y,z\right)=\prod \limits_{i=1}^n\left[{\omega}_{1\mid i}{f}_1\left({y}_i,{z}_i\right)+{\omega}_{2\mid i}{f}_2\left({y}_i,{z}_i\right)\right] $$

where *ω*_*j*|*i*_ is the conditional probability of QTL genotype *j* (*j* = 1 for *QQ* or 2 for *qq*), conditional on the marker interval that harbors the QTL, *f*_*j*_(*y*_*i*_,*z*_*i*_;**Σ**) is the bivariate density function of leaf area and leaf mass for QTL genotype *j*, and *Φ* represents the unknown parameters that describe the location of the QTL and its genetic effects on leaf allometry and residual (co)variances. The conditional probability is expressed in terms of the recombination fraction between the marker interval and QTL [52]. *f*_*j*_(*y*_*i*_,*z*_*i*_;**Σ**) is assumed to be a normal density function expressed as

$$ {f}_j\left({y}_i,{z}_i\right)=\frac{1}{2\pi {\sigma}_y{\sigma}_z\sqrt{1-{\rho}^2}}\exp \left[-\frac{1}{2\left(1-{\rho}^2\right)}\left[\frac{{\left({y}_i-{\mu}_{jy}\right)}^2}{\sigma_y^2}-2\rho \frac{\left({y}_i-{\mu}_{jy}\right)\left({z}_i-{\mu}_{jz}\right)}{\sigma_y{\sigma}_z}+\frac{{\left({z}_i-{\mu}_{jz}\right)}^2}{\sigma_z^2}\right]\right] $$

where *μ*_*jy*_ and *μ*_*jz*_ were defined as above and ${\sigma}_y^2$, ${\sigma}_z^2$ and *ρ* are the error variances of leaf area and leaf mass and their correlation coefficient, respectively.

The expectation-maximization (EM) algorithm was implemented to estimate the parameters *Φ* = (QTL position; *a*_*z*_, *b*, *β*; ${\sigma}_y^2$, ${\sigma}_z^2$, *ρ*). The significant QTL was estimated by assuming a QTL at every 2 cM position over the linkage map. After the parameters are estimated, we need to perform hypothesis tests. First, whether there exists a significant QTL can be tested by formulating the following hypotheses:

4 $$ {\mathrm{H}}_0:\cdot {\mu}_{1y}={\mu}_{2y}\cdot \mathrm{and}\cdot {\mu}_{1z}\cdot =\cdot {\mu}_{2z} $$

H_1_: At least one of the equalities in the H_0_ does not hold.

The log-likelihood ratio calculated from the H_0_ and H_1_ is compared against the genome-wide critical threshold determined from permutation tests. Second, we can test whether *β* is significantly different from a specific value, e.g. 1 or 3/4. The allometry theory can be used to interpret the biological meaning of these parameters [17].

#### Mapping ontogenetic allometry

Ontogenetic allometry states that leaf area (*A*) is scaled with leaf mass (*M*) over developmental time (*t*). Previous studies noted that as leaves grow, increases in surface area and mass are not synchronous [8]. To accommodate this phenomenon, we introduced an intercept *d* into the power equation [53], obtaining

5 $$ A(t)={\alpha M}^{\beta }(t)-\mathrm{d} $$

Let *Y*_*i*_(*t*) and *Z*_*i*_(*t*) denote the observed values of leaf area and leaf mass at time *t* (*t* = 1, …, *T*) for RIL *i* (*i* = 1, …, *n*), respectively. By statistical reasoning, we found a goodness-of-fit of eq. (5) to *Y*_*i*_(*t*) and *Z*_*i*_(*t*) data for individual RILs (Additional file 4).

To map how a QTL affects the ontogenetic allometry of leaf area vs. leaf mass, we implemented Zhao et al.’s [54] bivariate functional mapping. Consider a QTL with two genotypes *QQ* and *qq*. Let **Y**_*i*_ = (*Y*_*i*_(1), …, *Y*_*i*_(*T*)) and **Z**_*i*_ = (*Z*_*i*_(1), …, *Z*_*i*_(*T*)). The bivariate functional mapping is formulated as

6 $$ L\left(\varPhi |Y,Z\right)=\prod \limits_{i=1}^n\left[{\omega}_{1\mid i}{f}_1\left({Y}_i;{Z}_i\right)+{\omega}_{2\mid i}{f}_2\left({Y}_i;{Z}_i\right)\right] $$

where *ω*_*j*|*i*_ was defined as above, and *f*_*j*_(**Y**_*i*_; **z = Z**_*i*_) is a bivariate longitudinal normal distribution function for QTL genotype *j* (*j* = 1 for *QQ* and 2 for *qq*). The genotype-dependent mean vector of *f*_*j*_(**Y**_*i*_; **Z**_*i*_) is expressed as

7 $$ \left({\boldsymbol{\mu}}_{jy};{\boldsymbol{\mu}}_{jz}\right)=\left({\mu}_{jy}(1),\dots, {\mu}_{jy}(T);{\mu}_{jz}(1),\dots, {\mu}_{jz}(T)\right) $$

Based on the allometry law expressed by the power eq. (5), we model the genotypic value of leaf area by

8 $$ {\mu}_{jy}(t)={\alpha}_j{\upmu}_{jz}^{\beta_j}(t)-{d}_j $$

Based on the principle of functional mapping, we model the genotype-dependent growth of leaf mass *μ*_*jz*_(*t*) by a growth equation, such as logistic equation [55]. Thus, we have

9 $$ {\mu}_{jz}(t)=\frac{A_j}{1+{B}_j{e}^{- Rt}} $$

where parameters (*A*_*j*_, *B*_*j*_, *R*_*j*_) are the asymptotic growth, initial growth and relative growth rate of leaf mass for genotype *j* over time, respectively. Therefore, by substituting eqs. (8) and (9) into the mean vector (7), we can model the genotypic values of each QTL genotype for leaf area and leaf mass at different time points through parameters (*A*_*j*_, *B*_*j*_, *R*_*j*_; *α*_*j*_, *β*_*j*_, *d*_*j*_). Such modeling has incorporated the allometric scaling law and growth law, which makes QTL mapping of biological relevance and robustness.

The (co)variance matrix (**Σ**) of *f*_*j*_(**Y**_*i*_; **Z**_*i*_) is a symmetric matrix containing three longitudinal (co)variance matrices within and between leaf area and leaf mass. Zhao et al. [54] derived a bivariate first-order structured antedependence (bi-SAD(1)) model to fit the structure of **Σ**. The bi-SAD (1) model requires the following parameters: antedependence parameters (*ρ*_*y*_ and *ρ*_*z*_) and innovation variances ($$ {\sigma}_y^2 $$ and $$ {\sigma}_z^2\Big) $$ for leaf area and leaf mass, and correlation between the two traits (*ρ*_*yz*_). Based on these parameters, Zhao et al. [54] derived the closed forms of the determinants and inverse of the bi-SAD(1)-structured longitudinal matrix. By implementing such closed forms into the likelihood (6), we can greatly increase the computational efficiency and precision of parameter estimation *Φ* = (QTL position; *A*_*j*_, *B*_*j*_, *R*_*j*_; *α*_*j*_, *β*_*j*_, *d*_*j*_; *ρ*_*y*_, *ρ*_*z*_, *ρ*_*yz*_, $$ {\sigma}_y^2, $$$$ {\sigma}_z^2\Big) $$.

Accordingly, the estimation of unknown parameters can be made by a hybrid between the EM and simplex algorithms. The QTL position can be estimated by a grid approach. The presence and location of an ontogenetic-allometry QTL can be tested through the null hypotheses:

10 $$ {\mathrm{H}}_0:\left({A}_j,{B}_j,{R}_j;{\alpha}_j,{\beta}_j,{d}_j\right)\equiv \left(A,B,R;\alpha, \beta, d\right)\ \mathrm{for}\ \mathrm{all}\ j=1,2 $$

These tests are carried out by calculating the log-likelihood ratio for the QTL effect at each position and comparing it to the genome-wide critical threshold determined from permutations tests. We can specifically test whether the QTL affects the growth trajectory of leaf mass (11), and/or its ontogenetic allometry with leaf area (12).

11 $$ {\mathrm{H}}_0:\left({A}_j,{B}_j,{R}_j\right)\equiv \left(A,B,R\right)\ \mathrm{for}\ \mathrm{all}\ j=1,2 $$

12 $$ {\mathrm{H}}_0:\left({\alpha}_j,{\beta}_j,{d}_j\right)\equiv \left(\alpha, \beta, d\right)\ \mathrm{for}\ \mathrm{all}\ j=1,2 $$

If both null hypotheses are rejected, then this means that the QTL pleiotropically affects leaf area and leaf mass growth. Possible functions of all the QTLs were annotated via BLAST in the “nr” database on website of National Center of Biotechnology Information (NCBI; http://blast.ncbi.nlm.nih.gov/).

## Supplementary information

**Additional file 1.** The profile of log-likelihood ratio (LR) test statistics over 11 chromosomes for testing the existence of QTLs governing leaf area vs. leaf mass static allometry at different time points 1–5 for the common bean grown at Palmira (red) and Popayan (blue), with the LR values besides the most significant QTLs. The slash horizontal line denotes the genome-wide critical threshold determined from 1000 permutation tests.

**Additional file 2.** The profile of log-likelihood ratio (LR) test statistics over 11 chromosomes for testing the existence of QTLs governing leaf area vs. leaf mass ontogenetic allometry for the common bean grown at Palmira (red) and Popayan (blue), with the LR values besides the most significant QTLs. The slash horizontal line denotes the genome-wide critical threshold determined from 1000 permutation tests.

**Additional file 3.** The function annotation of all the QTLs, marker information and the genetic and environmental effects.

**Additional file 4 **The ontogenetic allometry fitting (curve) of leaf area and leaf mass data (dots) at 5 tome points for each RIL grown at Palmira (red) and Popayan (blue) by the intercepted power eq. (5) *A*(*t*) *= αM*^*β*^(*t*) – *d*.

## Data Availability

All data generated or analyzed during this study are included in this published article and its additional files.

## Abbreviations

QTL: Quantitative trait locus
SLA: Specific leaf area
LMA: Leaf mass per area
LOA: Leaf ontogenetic allometry
LSA: Leaf static allometry
A: Leaf area
M: Leaf mass
RIL: Recombinant inbred line
bi-SAD(1): Bivariate first-order structured antedependence
PAL: Palmira
POP: Popayan
CC: Calima-inherited genotype
JJ: Jamapa-inherited genotype
