# Supplementary figures and images for: The genetic control of leaf allometry in the common bean, Phaseolus vulgaris
Source: BMC Genet. 2020 Mar 14;21:29. doi: 10.1186/s12863-020-00838-2 (PMC7071654; doi:10.1186/s12863-020-00838-2)

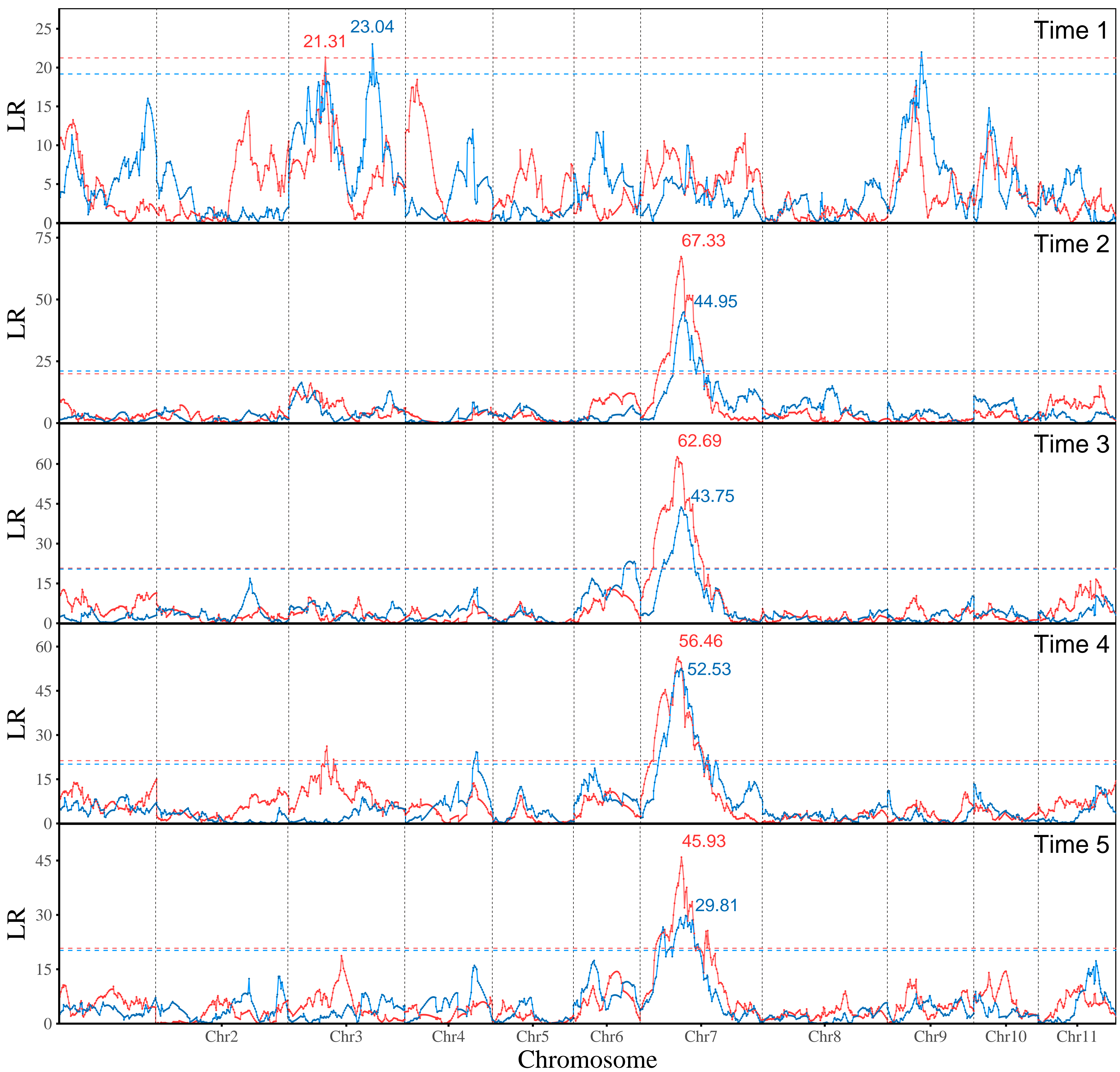

Supplement: Supplementary file 1 — Additional file 1. The profile of log-likelihood ratio (LR) test statistics over 11 chromosomes for testing the existence of QTLs governing leaf area vs. leaf mass static allometry at different time points 1–5 for the common bean grown at Palmira (red) and Popayan (blue), with the LR values besides the most significant QTLs. The slash horizontal line denotes the genome-wide critical threshold determined from 1000 permutation tests. [file 12863_2020_838_MOESM1_ESM.pdf]

LR

200  
150  
100  
50  
0

Chr2

Chr3

Chr4

Chr5

Chr6

Chr7

Chr8

Chr9

Chr10

Chr11

chromosome

198.25

154

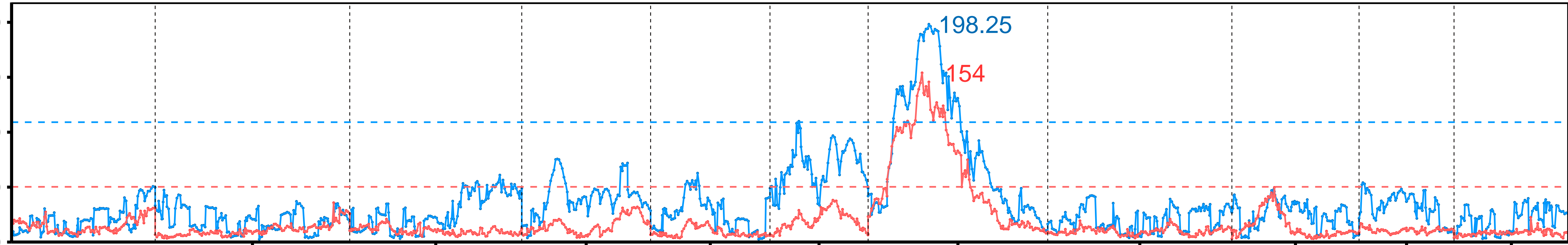

Supplement: Supplementary file 2 — Additional file 2. The profile of log-likelihood ratio (LR) test statistics over 11 chromosomes for testing the existence of QTLs governing leaf area vs. leaf mass ontogenetic allometry for the common bean grown at Palmira (red) and Popayan (blue), with the LR values besides the most significant QTLs. The slash horizontal line denotes the genome-wide critical threshold determined from 1000 permutation tests. [file 12863_2020_838_MOESM2_ESM.pdf]

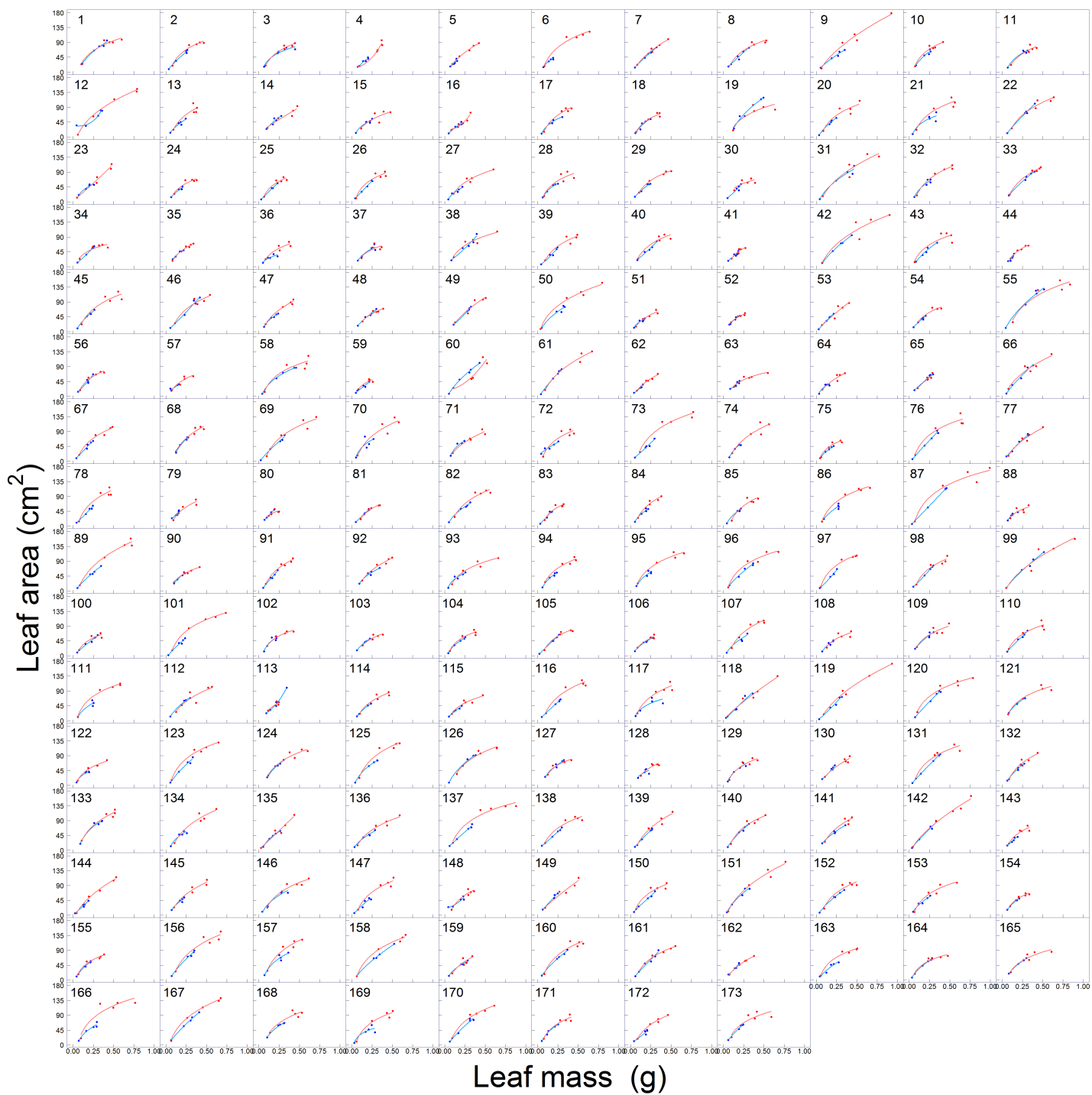

Supplement: Supplementary file 4 — Additional file 4 The ontogenetic allometry fitting (curve) of leaf area and leaf mass data (dots) at 5 tome points for each RIL grown at Palmira (red) and Popayan (blue) by the intercepted power eq. (5) A(t) = αMβ(t) – d. [file 12863_2020_838_MOESM4_ESM.pdf]
